# Mental health in sexual minorities: Change over time in a Finnish population-based sample

**DOI:** 10.1017/S0033291725102626

**Published:** 2025-11-18

**Authors:** Marianne Källström, Ida E. M. Pedersen, Daniel Ventus, Annika Gunst, Martin Lagerström, Sabina Nickull, Patrick Jern

**Affiliations:** 1 Psychology, The Faculty of Human and Social Sciences, Åbo Akademi University, Turku, Finland; 2 Experience Lab, Åbo Akademi University, Vaasa, Finland

**Keywords:** anxiety, alcohol, depression, sexual distress, sexual minority

## Abstract

**Background:**

Sexual minorities have continuously been found to experience poorer mental health compared to the general population, despite promising changes in attitudes and legislation throughout the 21st century in many Western countries. The present study is one of the first to assess group-level changes over time in mental health among sexual minorities compared to their heterosexual counterparts.

**Methods:**

We used four waves of a Finnish population-based survey spanning 16 years (2006–2022) to compare heterosexual and sexual minority adults on depression and anxiety symptoms, alcohol use, and sexual distress.

**Results:**

Sexual minority individuals reported more depression and anxiety symptoms, sexual distress, and alcohol use relative to their heterosexual counterparts at all time points. There were no group differences in the direction or rate of change in group means from 2006 to 2022. Depression and anxiety symptoms showed equally large increases, and alcohol use showed equally large decreases among both heterosexual and sexual minority participants.

**Conclusions:**

Contrary to our expectations based on minority stress theory, differences in mental health between sexual minority and heterosexual individuals persist despite changes in the sociolegal status of sexual minorities during the first two decades of the 21st century. Our findings align with the increasing general trend in anxiety and depression symptoms, which seems to affect the whole population regardless of sexual orientation. We conclude that the effect of legislative societal improvements seems to be small, and the mental health gap between sexual minority and heterosexual adults is likely maintained by factors not included in our study.

## Introduction

Many cross-sectional studies from the past two decades have found poorer outcomes on many mental health-related measures, such as depression, anxiety, and substance use among sexual minority (SM) individuals compared to their heterosexual counterparts (e.g. Lucassen et al., 2017; Wittgens et al., 2022). These discrepancies also encompass distress related to sexuality and one’s sex life, as SM people have also reported higher levels of sexual distress (Björkenstam, Mannheimer, Löfström, & Deogan, 2020; Burri & Spector, 2011). Simultaneously, improvements to SM’s sociolegal status have taken place in many Western countries. Based on theoretical perspectives such as the minority stress theory (Meyer, 1995, 2003), sociolegal status improvements should contribute to diminishing group differences in mental health based on sexual orientation; however, few longitudinal studies have investigated this.

## Depression and anxiety

Depression and anxiety are common mental disorders with severe adverse individual and societal impacts (World Health Organization, 2017). The prevalence of both depression (Moreno-Agostino et al., 2021) and anxiety (Goodwin et al., 2020) symptoms has increased during the last two decades, but few studies have investigated changes over time among SMs. Bränström and Pachankis (2018) compared levels of psychological distress in a Swedish population-based survey and found a decrease in distress among lesbian and gay participants compared to heterosexual participants over 10 years (2005–2015). By contrast, a longitudinal study with heterosexual and SM adolescents in the United States conducted by Parodi et al. (2022) found more rapidly increasing anxiety levels among SM youth compared to heterosexual youth from 2012 to 2018.

When estimating population change over time in mental health, it is possible that any observed changes reflect true change among the population studied, but variation over time may also depend on age-related reasons, such as cohort effects (Jorm, 2000). Younger cohorts of SM adults have reported poorer mental health than older cohorts (Mallory, Russell, & Meyer, 2023; Meyer et al., 2021; Russell et al., 2022) and differences between SM and heterosexual adults have been more pronounced in younger cohorts (Liu & Reczek, 2021). Nevertheless, older SM adults remain at increased risk of psychological distress compared to their heterosexual counterparts (Fredriksen-Goldsen et al., 2013). Another epidemiological trend related to the question of temporal changes in SM mental health is the rise in self-reported SM identities during the 21st century (IPSOS, 2025; Twenge, Sherman, & Wells, 2016: Twenge, Wells, & Le, 2024). Recent studies have found sexual identity shifts toward a more same-sex oriented direction to be associated with increased depression and anxiety symptoms in young adults and adolescents (Campbell et al., 2022; Xu & Rahman, 2024). Identity change may be inherently stressful, and especially change in a more same-sex oriented direction can be hypothesized to introduce new stressors into one’s life as one might experience or anticipate prejudice or discrimination in situations where one has not previously done so.

## Alcohol use

Alcohol misuse is a serious and common problem, with 5% of worldwide annual deaths caused by alcohol-related diseases (World Health Organization, 2024). According to previous reviews, SMs are at particular risk for alcohol misuse (King et al., 2008; Lewis, 2009; Plöderl & Tremblay, 2015) and alcohol use disorder (Wittgens et al., 2022). Longitudinal studies on SMs have found an initial increase in alcohol consumption from adolescence to young adulthood (likely influenced by reaching legal drinking age; Becker, Cortina, Tsai, & Eccles, 2014; Dermody et al., 2014; Marshal et al., 2012), followed by stabilization (Becker et al., 2014; Dermody et al., 2014) and a steady decrease throughout adulthood (Boehmer, Miao, Linkletter, & Clark, 2012; Talley et al., 2023). Studies including heterosexual comparison groups have found greater initial increases among young SM adults compared to older SM adults (Becker et al., 2014; Boehmer et al., 2012; Dermody et al., 2014; Marshal et al., 2012). While the worldwide mean alcohol consumption has decreased during the 21st century (World Health Organization, 2024), it is unclear whether the same trend exists among SM individuals.

## Sexual distress

Sexual distress refers to negative feelings such as worry, frustration, or inadequacy regarding one’s sexual function or sexuality (Santos-Iglesias, Mohamed, & Walker, 2018; Stephenson & Meston, 2010). It is a diagnostic criterion for most sexual dysfunction diagnoses (World Health Organization, 2019), and is associated with poorer mental health (Burri, Rahman, & Spector, 2011; Hayes et al., 2008). Although often considered in relation to a specific sexual problem, more general assessments of sexual distress may capture distress stemming from other, unspecified aspects of sexual life (e.g. relationship factors; Santos-Iglesias et al., 2018). Such general sexual distress may be of particular interest when it comes to SMs, who may face unique stress and social stigma due to their sexuality (Meyer, 2003). Despite sexual distress receiving an increased amount of research attention during the 21st century, SMs remain largely unnoticed by these efforts. According to previous studies, people who are bisexual or report same-gender sexual experiences have more sexual distress than heterosexual people (Björkenstam et al., 2020; Burri & Spector, 2011), but we found no longitudinal studies exploring sexual orientation-based group differences over longer periods of time.

## Why do SMs experience poorer health outcomes?

The minority stress theory proposes that higher rates of mental health symptoms among SM individuals are due to unique minority stressors, such as experiences of discrimination, violence, and prejudice, often followed by subsequent vigilance and internalized homophobia (Meyer, 1995, 2003). The social climate surrounding SMs has undergone positive changes in the end of the 20th century and the beginning of the 21st century, such as diminishing prejudice and intolerance in many Western countries (European Union Agency for Fundamental Rights, 2024; Kenny & Patel, 2017) and improvements in SM-related laws and policies (ILGA World, 2024; PEW, 2024). Legislative improvements such as the introduction of same-sex marriage laws have been associated with positive general impacts on the physical health of SM people, but findings pertaining to mental health rely mostly on cross-sectional studies and feature some inconclusive results (Badgett, Carpenter, Lee, & Sansone, 2025; Drabble et al., 2021). For example, Eisner et al. (2025) found that while changes in same-sex marriage rights are associated with changed social norms in Western countries, such as Switzerland and Australia, no clear effect on mental health was distinguished. Increasing acceptance and legislative equality of SM people can still be hypothesized to lower minority stress and improve mental health among SM individuals, as it would be reasonable to expect that such structural changes diminish minority stress.

The reasons behind the larger burden of mental health problems among SM people may, however, be more complex than the minority stress theory proposes. Even though children who later adopt an SM identity experience more bullying and other vulnerability-increasing social stressors than heterosexual children, mental health discrepancies between the groups emerge earlier than differences in peer victimization (Booth & Fitzsimons, 2025). The connection between SM status and mental health has been hypothesized to be partly explained by shared genetic influences affecting both sexual orientation and mental health independently (Ganna et al., 2019) or by a mix of genetic influences and phenotypic causal pathways (Oginni et al., 2023). For example, when researchers have controlled for child abuse (Xu & Rahman, 2022) and gender nonconformity (Oginni, Alanko, Jern, & Rijsdijk, 2024), associations between SM status and mental health problems diminish in magnitude. The question of what kind of a role of minority stress plays in relation to other genetic, developmental, and psychological factors is therefore still unresolved.

As mental health symptomatology has also increased in the general population (World Health Organization, 2017), this raises the question of how mental health among SM populations stands in relation to mental health among their heterosexual counterparts. We set out to explore changes in mental health among the SM and heterosexual population in Finland by investigating symptoms of depression, anxiety, alcohol use, and sexual distress in a population-based sample of heterosexual and SM individuals at four time points between 2006 and 2022. We formed four hypotheses (H1–H4) and one exploratory research question (Q1):

(H1) Heterosexual individuals will report lower levels of depression and anxiety symptoms, alcohol use, and sexual distress than SM individuals at the first time point (2006).

(H2) Differences between SM and heterosexual individuals regarding symptoms of depression and anxiety, alcohol use, and sexual distress will show a decreasing trend from 2006 to the last time point (2022).

(H3) The level of depression and anxiety symptoms will increase between 2006 and 2022 in both groups.

(H4) The level of alcohol consumption will decrease between 2006 and 2022 in both groups.

(Q1) How has the level of sexual distress changed in both groups between 2006 and 2022?

## Methods

### Participants

The present study was based on four waves of a population-based survey known as the Genetics of Sex and Aggression project. Participants consisted of Finnish twins and their siblings and parents aged 18 and older. Data were collected in 2006 (T1), 2012–2013 (T2), 2018–2019 (T3), and 2021–2022 (T4). Please see Table 1 for an overview of the sample characteristics in each wave and the Supplement for a description of the data collection procedure.

**Table 1.** Procedures and sample descriptives for the four waves of the genetics of sex and aggression data collection

	T1 (2006)	T2 (2012–2013)	T3 (2018–2019)	T4 (2021–2022)
Target sample	All Finnish twins aged 18–33 and their biological siblings	T1 participants who stated interest in further participation	All Finnish twins aged ≥18 and their biological siblings	All Finnish twins aged ≥18, their biological siblings and parents
*N* contacted	23,577	7,756	33,211	50,771
*N* responded	10,524	3,346	9,564	12,269
Response rate^a^	45 %	43 %	29 %	24 %
% participated previously	N/A	100 %	30 %	34 %
Survey method	Paper or online	online	online	online
*n* twins	6,531	2,324	6,818	4,841
*M(SD)* age	24.97 (4.01)	35.76 (5.90)	29.04 (7.69)	31.14 (7.53)
Range (min–max)	18–33	24–59	18–27	20–48
*n* siblings	3,993	871	2,501	1,807
*M(SD)* age	28.58 (5.97)	32.17 (5.71)	32.82 (8.65)	33.91 (8.92)
Range (min–max)	18–49	23–56	18–44	18–64
*n* mothers	—^b^	—^b^	—^b^	3,138
*M(SD)* age	—^b^	—^b^	—^b^	61.13 (7.25)
Range (min–max)	—^b^	—^b^	—^b^	41–86
*n* fathers	—^b^	—^b^	—^b^	1,629
*M(SD)* age	—^b^	—^b^	—^b^	63.76 (7.35)
Range (min–max)	—^b^	—^b^	—^b^	43–88
*N* included in present study	7,023	3,194	9,319	11,415

*Note*: The participants were Finnish twins and siblings of twins, whose contact information were obtained from the Finnish Central Population Registry. The participants were approached by postal mail (*N* contacted) and asked to participate in the survey.

a Response rate is inclusive of individuals with missing values or who declined participation.

b Parents of the twins were not included in the sample in T1–T3.

### Ethical standards

All procedures contributing to this study comply with the ethical standards of the Finnish National Board on Research Integrity and the Ethics Review Board of Åbo Akademi University, as well as the Helsinki Declaration of 1975, as revised in 2008. The research plan was reviewed by the Ethics Review Board of Åbo Akademi University and received approval prior to data collection.

### Measures

#### Mental health

Symptoms of depression and anxiety were measured using the depression (Cronbach’s α = .84–.87 depending on time point) and anxiety (Cronbach’s α = .85–.87) subscales of the Brief Symptom Inventory-18 (BSI-18; Derogatis & Melisaratos, 1983). Sum scores were calculated for each subscale, with higher scores indicating more symptoms (range: 6–30). We measured alcohol use with the three-item concise version of the Alcohol Use Disorders Identification Test (AUDIT-C; Bush et al., 1998), which is an effective tool for predicting hazardous drinking (Tuunanen, Aalto, & Seppä, 2007). We calculated sum scores, with higher scores indicating more alcohol use (range: 0–12; Cronbach’s α = .57–.62). We measured sexual distress with an abbreviated gender-neutral seven-item version of the Sexual Distress Scale (SDS; Derogatis et al., 2002; for validation in male samples see, e.g. Tavares, Santos-Iglesias, & Nobre, 2022). We formed sum scores for which higher scores indicated more distress (range: 7–35; Cronbach’s α = .89–.92).

#### Sexual orientation

Although sexual orientation was assessed at all time points (see Supplement), we used the T4 sexual orientation answer as our grouping variable for our analyses, as it provided the most recent information. The T4 sexual orientation was based on a multiple-choice question with the options ‘heterosexual’, ‘lesbian/gay’, ‘bisexual’, ‘pansexual’, ‘asexual’, and ‘other’. Those who selected heterosexual were categorized as heterosexual, and all others as SM participants.

### Statistical analyses

We excluded individuals with >50% missingness on any measure from the analyses of that measure. Individuals with ≤50% missingness had missing observations imputed using intrascale information with the Expectation Maximization procedure in SPSS 28.0. After imputation, we had complete responses from *N*_T1_= 7,023, *N*_T2_= 3,194, *N_T3_* = 8,593, *N_T4_* = 9,745 individuals on the BSI-18, *N_T1_* = 6,336, *N_T2_* = 2,976, *N_T3_* = 7,771, *N_T4_* = 8,519 individuals on the AUDIT-C, and *N_T1_* = 6,971, *N_T2_* = 3,195, *N_T3_* = 9,120, *N_T4_* = 9,499 individuals on the SDS. These were merged into a combined T1–T4 sample (*n* = 21,323), from which 22 participants with unclear or inadequate answers on sexual orientation or gender identity were omitted due to data-quality concerns (e.g. ‘attack helicopter’, see Supplement for further information). From the remaining participants (*n* = 21,301), we selected all those fulfilling the following criteria: (a) participated in T4 and reported their sexual orientation in the T4 survey (*n* = 11,125) and (b) had completed at least one outcome measure at any time point. This resulted in a sample of 10,094 participants, which consisted of both a longitudinal subsample of individuals who participated on T1, T2, and/or T3 as well as T4, and a cross-sectional T4 sample (for details on the different subsamples, please see Supplementary Table S1).

We conducted analyses of change over time in the SM and heterosexual samples by fitting latent growth curve models (LGCMs) in MPlus version 8.8. We selected LGCM because they allowed us to examine the initial levels of the outcome variables (i.e. intercepts), their rate and direction of change (i.e. slopes), as well as individual variance in the two, and the association between intercept and slope (i.e. whether a higher or lower initial outcome score is associated with a certain direction or rate of change). Sexual orientation at T4 was selected as a grouping variable. The T1 outcome variable score was set as baseline, and the unit of time was set to 1 year, meaning the slope variable reflected the annual outcome score change. Standard errors and chi-square tests of model fit were calculated using a sandwich estimator to account for clustering in the data due to genetic relatedness between participants from the same family. All participants were given a family code during data collection, which was the same for participants within the same family. This way, the fact that a subset of the sample consisted of genetically interrelated individuals (i.e. twins, parents, and fraternal siblings) could be controlled for in our analyses. This resulted in *N* = 6,144 (depression and anxiety models), *N* = 5,669 (alcohol-use model), and *N* = 6,029 (sexual-distress model) clusters in the heterosexual groups. The corresponding cluster sizes for the SM group were *N* = 1,005, *N* = 889, and *N* = 998. Slope variance was fixed at zero in the SM group for models of depression, anxiety, and sexual distress due to issues of negative variance values (i.e. Heywood cases). Group differences in baseline scores and change over time were assessed by fitting new models in which the intercepts and slopes were forced to be equal in the SM and heterosexual group, followed by tests of model fit via Wald χ^2^-test to indicate whether model fit was significantly worse compared to the original models.

The proportion of SM participants in the final sample used in the LGCMs was 9.4–18.7%, which meant that for any given outcome measure at any given time point, the number of SM participants ranged from 112 to 1,057 (see Supplementary Table S3). The combined sum of SM participants was sufficient to allow us to estimate the abovementioned models comparing heterosexual and SM individuals. However, modeling subgroup changes in different SM orientations (e.g. gay, lesbian, bisexual) or across different age groups was not feasible considering the sample sizes needed to reliably model changes over time using LGCM (Hamilton, Gagné, & Hancock, 2003; Preacher, 2018).

As the *p*-value of the χ^2^ statistic is affected by sample size and therefore not always appropriate for use in large samples, we used the root-mean-square error of approximation (RMSEA; cut-off value ≤.08), the comparative fit index (CFI; cut-off value ≥ .90), the Tucker–Lewis index (TLI; cut-off value ≥.90), and the standardized root-mean-square residual (SRMR; cut-off value ≤ .08) as additional fit indices to determine model fit (Hu & Bentler, 1999).

## Results

The final sample (*n* = 10,094) had a mean age of 26.02 years (*SD* = 4.88) at T1, 33.32 years (*SD* = 4.97) at T2, 30.84 years (*SD* = 8.27) at T3, and 44.14 years (*SD* = 16.55) at T4. Descriptive statistics on sex as recorded in the Central Population Registry, gender identity, and sexual orientation are presented in Table 2. Descriptive data on the number of participants who were classified as heterosexual at one time point and as an SM at another can be found in the Supplement. Means and standard deviations for all outcome measures are reported in Table 3.

**Table 2.** Participant characteristics in the final sample used for the growth curve models

Characteristic	T1		T2		T3		T4	
	*n*	%	*n*	%	*n*	%	*n*	%
Sex^a^								
Man	372	22.61	372^b^	30.57	830	23.83	3,329	32.98
Woman	1,273	77.39	845^b^	69.43	2,653	76.17	6,765	67.02
Gender Identity^c^								
Cisgender	1,633	99.27	1,211	99.26	3,418	98.13	9,981	98.88
Gender Minority	12	0.73	9	0.74	65	1.87	113	1.12
Sexual Orientation^d^								
Heterosexual	1,489	90.52	1,100	90.16	2,844	81.65	8,987	89.03
Lesbian/Gay	42	2.55	39	3.20	113	3.24	205	2.03
Bisexual	81	4.92	60	4.29	343	9.85	592	5.86
Pansexual	21	1.28	13	1.07	95	2.73	153	1.52
Asexual	7	0.43	5	0.41	57	1.64	92	0.91
Other	5	0.30	3	0.25	31	0.89	65	0.64
Total SM	156	9.48	120	9.84	639	18.35	1,107	10.97

*Note: N* = 1,645 at T1 (2006). *N* = 1,220 at T2 (2012/2013). *N* = 3,483 at T3 (2018/2019). *N* = 10,094 at T4 (2021/2022). SM = sexual minority.

a Sex as recorded in the Central Population Registry.

b Sex was not recorded at T2 but derived from T1, as T2 was a subsample of T1, *n* = 3 missing.

c Gender identity as reported at T4.

d Sexual orientation as reported at T4.

**Table 3.** Means, standard deviations, and sample sizes for the outcome variables for the four time points

Time point	Depression^a^			Anxiety^a^			Alcohol use^b^			Sexual distress^c^		
	*n*	*M*	*SD*	*n*	*M*	*SD*	*n*	*M*	*SD*	*n*	*M*	*SD*
	Heterosexual											
T1	1,475	10.65	4.29	1,475	9.43	3.76	1,349	4.72	2.25	1,468	13.74	5.26
T2	1,078	10.17	4.33	1,078	8.91	3.73	1,007	4.62	2.20	1,078	14.33	5.92
T3	2,740	11.87	5.06	2,740	10.52	4.56	2,512	4.25	2.05	2,821	14.43	5.53
T4	8,688	11.63	4.58	8,688	10.48	4.26	7,626	3.94	2.12	8,456	13.58	5.75
	Sexual minority											
T1	153	11.69	4.49	153	10.61	4.56	139	4.96	2.27	152	14.14	5.31
T2	120	10.47	4.66	120	9.67	4.12	112	5.06	2.23	120	14.16	5.36
T3	632	14.15	5.84	632	12.60	5.53	544	4.47	2.12	638	15.02	5.89
T4	1,057	14.56	5.70	1,057	13.48	5.61	893	4.17	2.24	1,043	14.93	6.47

*Note: N* = 10,094. T1 = Time point 1 (2006). T2 = Time point 2 (2012/2013). T3 = Time point 3 (2018/2019). T4 = Time point 4 (2021/2022). SM = sexual minority.

a *N* = 10,081. Range = 6–30.

b *N* = 9,036. Range = 0–12.

c *N* = 9,884. Range = 7–35.

**Figure 1.**
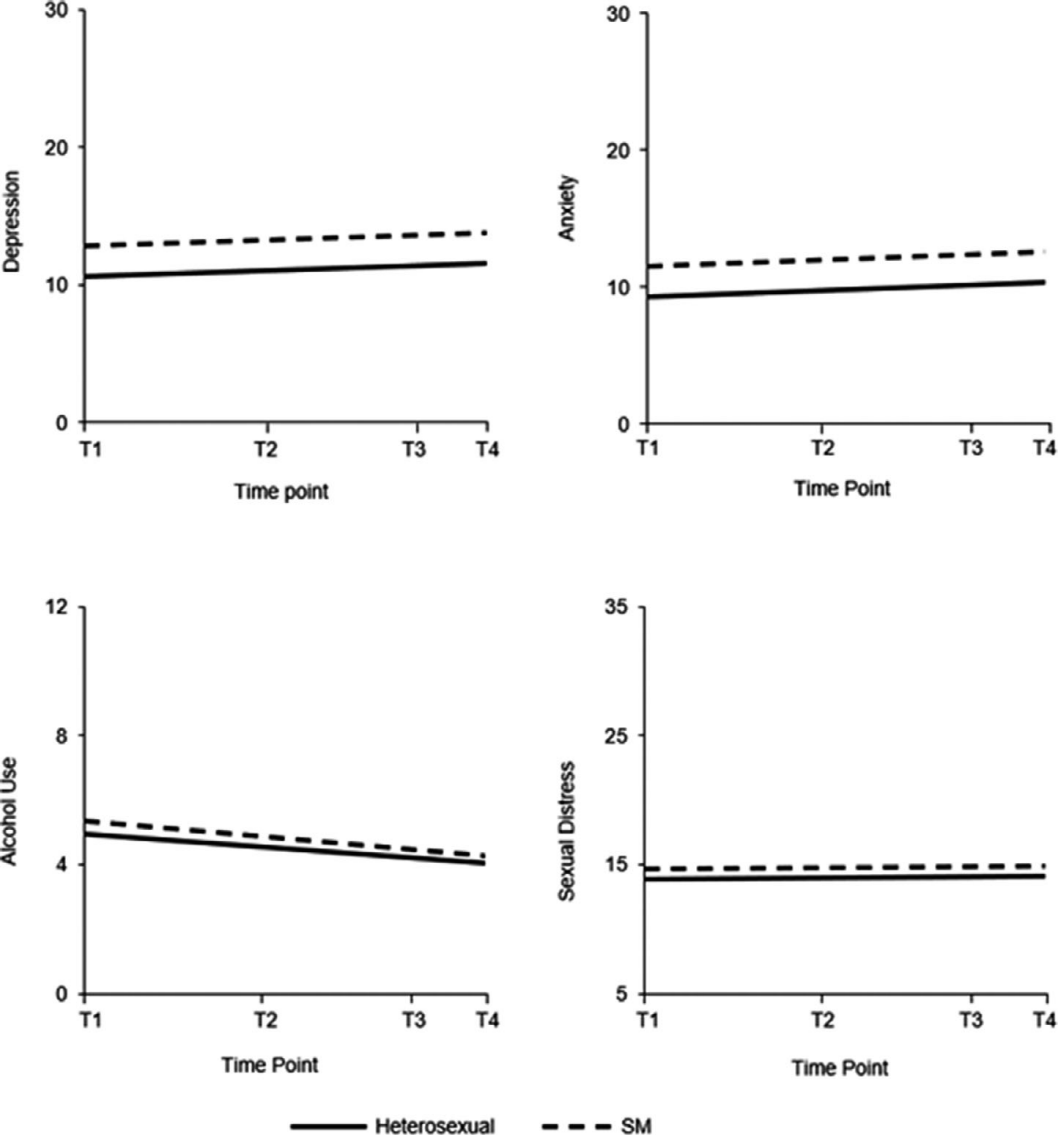
Graphical representation of changes in depression and anxiety symptoms, alcohol use, and sexual distress from 2006 to 2022. *Note:* T1 = Time point 1 (2006). T2 = Time point 2 (2012/2013). T3 = Time point 3 (2018/2019). T4 = Time point 4 (2021/2022). Depression and anxiety score range = 6–30. Alcohol use score range = 0–12. Sexual distress score range = 7–35. SM = Sexual minority.

The model fit for depression was good (χ^2^[12] = 78.623, *p* < .001; RMSEA = 0.033, 90% CI [0.026–0.040], *p* = 1.000; CFI = 0.944; TLI = 0.944; SRMR = 0.080). In both the heterosexual and SM groups, there was a significant small increase in depression symptoms over time (see Figure 1 and Table 4). The SM group had a significantly higher baseline level of depression symptoms (Wald χ^2^[1] = 37.970, *p* < .001), but there was no significant difference between the groups in rate or direction of change over time. There were significant within-group differences in baseline depression scores in both groups, as indicated by the significant variance in intercepts. The slope variance in the heterosexual group was also significant, meaning that individuals within the group differed significantly in the amount or direction of change in depression scores over time. In the heterosexual group, there was no significant correlation between slope and intercept, indicating that participants’ depression scores changed at a similar rate regardless of the initial level of depression. As slope variance was fixed at zero in the SM group, no within-group differences in change over time or correlation between intercept and slope could be calculated. The model explained a significant proportion of the variance in both the heterosexual group (*R^2^* range across time points = .466–.601, all *p*-values < .001) and the SM group (*R^2^* range = .554–.594, all *p*-values <.001).

**Table 4.** Means and standard errors for intercepts and slopes

	Depression		Anxiety		Alcohol use		Sexual distress	
	Heterosexual	SM	Heterosexual	SM	Heterosexual	SM	Heterosexual	SM
Intercept								
*M*	10.625	12.837	9.290	11.555	4.936	5.354	13.851	14.622
*SE*	0.110***	0.346***	0.093***	0.311***	0.055***	0.164***	0.132***	0.360***
Slope								
*M*	0.063	0.106	0.072	0.108	−0.062	−0.073	−0.010	0.023
*SE*	0.007***	0.023***	0.006***	0.020***	0.003***	0.011***	0.009	(0.025)
Variance: Intercept								
*M*	9.948	18.416	6.630	17.912	3.476	3.339	11.077	16.322
*SE*	1.200***	1.338***	0.984***	1.458***	0.208***	0.655***	1.605***	1.510***
Variance: Slope								
*M*	0.024	—^a^	0.012	—^a^	0.009	0.012	0.049	—^a^
*SE*	0.006***	—^a^	0.005*	—^a^	0.001***	0.004**	0.009***	—^a^
Correlation coefficient^b^	−0.214	—^a^	.102	—^a^	−0.375	−0.384	−0.192	—^a^
*SE*	0.117	—^a^	0.234	—^a^	0.046***	0.135**	0.124	—^a^

*Note. N* = 10,081 for depression and anxiety. *N* = 9,036 for alcohol use. *N* = 9,884 for sexual distress. Slope means indicate score change in 1 year. * = *p* < .05. **= *p* < .01. *** = *p* < .001.

a Variance in slope fixed at zero.

b Refers to the correlation between intercept and slope.

The model fit for anxiety was good (χ^2^[12] = 77.974, *p* < .001; RMSEA = 0.033, 90% CI [0.026–0.040], *p* = 1.000; CFI = 0.946; TLI = 0.946; SRMR = 0.070). There was a significant but small increase in anxiety scores over time in both groups (see Figure 1 and Table 4). Following the same pattern as for depression, the SM group had a significantly higher baseline levels of anxiety (Wald χ^2^[1] = 49.240, *p* < .001), but there was no significant group difference in the amount or direction of change over time. There were also significant within-group differences in baseline anxiety in both groups, as well as differences in change over time in the heterosexual group. Slope variance was fixed at zero in the SM group. There was no significant correlation between slope and intercept in the heterosexual group, indicating that individuals’ anxiety scores changed in a similar manner over time regardless of baseline levels. The model explained a significant proportion of the variance in both the heterosexual group (*R^2^* range across time points = .457–.588, all *p*-values <.001) and the SM group (*R^2^* range = .572–.639, all *p*-values <.001).

The model fit for alcohol use was good (χ^2^[10] = 95.215, *p* < .001; RMSEA = 0.043, 90% CI [0.036–0.052], *p* = .902; CFI = 0.968; TLI = 0.961; SRMR = 0.034). There was a small significant decrease in alcohol use over time in both groups (see Figure 1 and Table 4). The SM participants had a significantly higher baseline alcohol use score (Wald χ^2^[1] = 5.864, *p* = .016), but the groups did not differ significantly in the rate or direction of change. The intercept and slope were significantly negatively correlated in both groups, indicating that the higher the initial alcohol use score, the more scores decreased over time. Both groups showed significant within-group differences in baseline alcohol use and change over time. The model explained a significant proportion of the variance in both the heterosexual group (*R^2^* range across time points = .663–.796, all *p*-values <.001) and the SM group (*R^2^* range = .573–.788, all *p*-values <.001).

The model fit for sexual distress was good (χ^2^[12] = 79.248, *p* < .001; RMSEA = 0.034, 90% CI [0.027–0.041], p = 1.000; CFI = 0.945; TLI = 0.945; SRMR = 0.060). Neither group showed significant changes over time (see Figure 1 and Table 4). The SM group had significantly higher baseline sexual distress levels (Wald χ^2^[1] = 3.976, *p* = .046), but the groups did not significantly differ in rate or direction of change. Within-group baseline scores varied significantly in both groups, as did change over time in the heterosexual group. Slope variance in the SM group was fixed at zero. The lack of a significant correlation between slope and intercept in the heterosexual group indicated that the rate and direction of change (or lack thereof) was not dependent on baseline scores. The model explained a significant proportion of the variance in both the heterosexual group (*R^2^* range across time points = .340–.573, all *p*-values <.001) and the SM group (*R^2^* range = .399–.487, all *p*-values <.001).

## Discussion

The present study assessed changes in mental health among SM individuals compared to heterosexual individuals in a Finnish population-based sample spanning over 16 years. We compared symptoms of depression and anxiety, alcohol use, and sexual distress, expecting to see both general and minority-specific changes over time. Heterosexual participants reported lower levels of depression and anxiety symptoms, sexual distress, and alcohol use at T1 (2006) compared to the SM participants, just as we expected. These results align with minority stress theory and with previous research (Bränström & Pachankis, 2018; Lucassen et al., 2017; Mattelin, Fröberg, Korhonen, & Khanolkar, 2022; Wittgens et al., 2022).

Our results also replicated previous findings concerning increasing levels of depression and anxiety and decreasing levels of alcohol use (Goodwin et al., 2020; Moreno-Agostino et al., 2021; World Health Organization, 2024). Contrary to our expectations, however, depression and anxiety symptoms increased at a similar rate among heterosexual and SM participants, meaning that no diminishing gap between the groups was detected. Alcohol use followed a similarly decreasing pattern in both groups, as group differences persisted even though both groups drank less. One potential explanation for the persistent group differences is that the changes in attitudes toward SM individuals have taken place too recently, meaning that more time may need to pass before measurable improvements in SM mental health may be observed. It might also be argued that while SM’s sociolegal status has improved, too many stressors remain unchanged (Meyer, 2016). For instance, while positive changes such as legal recognition of same-sex marriage have a positive impact on SM individuals (Chen & van Ours, 2022; Tatum, 2017), the public discussion surrounding SM rights may impact them negatively by increasing stress levels (Horne, McGinley, Yel, & Maroney, 2022).

Another possibility is that the effect of beneficial societal changes is relatively small compared to the impact of other potential factors, such as common genetic influences affecting both mental health and sexual orientation (Ganna et al., 2019; Zietsch et al., 2012). It is also possible that even though genetic mechanisms affect the connection between sexual orientation and mental health, they may do so through phenotypical causal pathways (Oginni et al., 2023). For example, the association between sexual orientation and mental health decreases slightly when controlling for early life adversities (Xu & Rahman, 2024) and even more so when controlling for childhood gender nonconforming behavior (Oginni et al., 2024). It is therefore possible that these factors, or some other previously unmeasured biological or social aspects, confound the association between sexual orientation and mental health. This does not mean that minority stress is not a relevant environmental factor, but rather that there might be considerable individual variability in the vulnerability to minority stressors or how strong one’s exposure to them is from an early age. For example, Oginni et al. (2024) concluded that the degree of childhood gender nonconforming behavior may play a crucial part in determining how likely one is exposed to childhood adversities such as a stressful parent–child relationship or peer victimization, which in turn heighten the risk for psychopathology in adulthood.

Some noteworthy characteristics of our sample may also have contributed to these results. The SM participants consisted of a majority of bisexual people. Bisexual individuals often experience worse mental health compared to lesbian and gay individuals (Hatzenbuehler, Bränström, & Pachankis, 2018; Hill et al., 2020; Spittlehouse, Boden, & Horwood, 2020), potentially due to unique minority stressors, such as double discrimination from both heterosexual and gay/lesbian communities (Colledge, Hickson, Reid, & Weatherburn, 2015; Dodge et al., 2016; Feinstein & Dyar, 2017). More accepting attitudes toward SMs may therefore favor lesbian and gay people over other SM subgroups. Future research should attempt to identify potential differences between SM identities in terms of how their mental health has changed over time.

Our sample also had a high proportion of SM participants at T3 (18.35%) relative to other time points (9.48%–10.97%). This may be partly explained by the relatively young mean age in the T3 sample (due to the influx of novel participants), as young people are more likely to report belonging to an SM (Gates, 2014). Younger SM individuals have also been known to report poorer mental health (Mallory et al., 2023; Meyer et al., 2021; Russell et al., 2022), which may in part have contributed to the higher means of depression and anxiety at T3 compared to earlier time points. However, participants of T4 had the highest mean age and were also found to report the highest mean symptom scores.

The fact that sexual identity reports are fluid in nature (Diamond, 2016) is also a factor to consider when comparing how groups of people change over time based on their sexual orientation. Reporting an SM identity when asked about one’s orientation has increased over time (Twenge et al., 2024), as has same-sex sexual behavior (Diamond, 2016; Twenge et al., 2016). Self-reported sexual identity and sexual behavior are nonetheless two separate ways of operationalizing sexual orientation, and they are strongly associated but do not fully overlap (Fu et al., 2019). Especially same-sex sexual behavior is common among heterosexual people, and especially women tend to report more same-sex sexual experience than men regardless of their stated sexual orientation (Diamond, 2016; Fu et al., 2019). Simply having engaged in same-sex sexual behavior does not necessarily have an association with psychological distress, whereas changing sexual identity is more clearly associated with distress. The association between sexual identity changes and mental health has been attributed to the inherently stressful nature of identity change, but recent studies have found depression and anxiety symptoms to increase only in people whose identity changed in a more same-sex oriented direction (Campbell et al., 2022; Xu & Rahman, 2024). In the present study, changes in sexual orientation among participants who took part in multiple time points were rare (see Supplement). An interesting topic for future studies would be to try to discern whether changes in distress only ensue in people who change their stated orientation and not in people whose sexual behavior changes without any corresponding identity change.

Future research may attempt to further discern potential changes in mental health over time among different age cohorts, as the number of SM participants in the present sample did not allow for including cohort-specific comparisons of change in SM and heterosexual individuals in our study. The question of cohort effects in the context of mental health and sexual orientation is relevant especially since both the prevalence of reporting psychological distress and reporting an SM identity have risen recently (Liu & Reczek, 2021; Twenge et al., 2016, 2024). The observed changes in these two phenomena may stem from different sources, or they could potentially be connected to, for example, changes in how people typically respond to survey questions on sex and mental health.

Sexual distress is the only measure for which we found no changes over time, and for which we found no prior hypothesis-generating research. The level of sexual distress remained stable in both groups, with no signs of a diminishing gap between heterosexual and SM individuals. In light of the associations between sexual distress and more general psychological distress such as depression (see, e.g. Dennerstein et al., 2008), it is somewhat surprising that sexual distress remained stable as anxiety and depression levels increased. There was significant variance in the intercepts for sexual distress, suggesting within-group differences in baseline sexual distress within both the SM and heterosexual groups. This means that grouping all SMs together may have failed to capture changes in specific sexual-identity groups. Our sample on SM participants was not large enough to allow us to explore our sample at this level of detail, but potential changes over time in sexual distress among specific SM groups would be an interesting question for further research.

### Strengths and limitations

To our knowledge, the current study is the largest and longest spanning one investigating how mental health in SMs has changed during the beginning of the 21st century, when positive changes in societal attitudes and legislation around SM issues have taken place in many Western countries. We utilized a population-based sample, which can be considered more representative than many prior samples. Although minority groups make up a small portion of a population-based sample, this type of data nevertheless has the potential to include minority individuals who may be missed by clinical and community samples (e.g. people without clinical diagnoses and people who are not active in local LGBTQ communities).

Even though our study provides valuable and novel information about changes over time in mental health among SM individuals in general, we did not have a sufficiently large sample of SM participants to further explore differences between specific SM groups. Another limitation is that our data did not include direct measures of minority stressors, such as experiences of discrimination, so modeling potential mediating or moderating factors derived from minority stress theory was not possible. Considering the scarcity of similar research focusing on temporal changes in SM mental health, we believe that our results nonetheless offer a valuable contribution to this research field.

We found significant variance in both baseline scores and change over time (in the instances it was calculated) in all outcome variables and both groups, indicating that many factors apart from sexual orientation influenced the outcome variables. The LGCMs also included participants who did not participate at every time point, which contributed to low covariance coverage between earlier and later time points, potentially increasing estimation difficulty. This may have contributed to Heywood cases in the SM group LGCMs, or the variance may truly be close to zero.

## Conclusions

People who belong to SMs reported worse mental health at all time points in our study (i.e. between 2006 and 2022). Contrary to the expectations we formed based on minority stress theory, we detected no signs of a diminishing mental health gap between the groups. Symptoms of depression and anxiety increased instead to a similar degree in both SM and heterosexual individuals, and the mean level of alcohol use decreased to a similar degree in both groups. These results call into question the idea that improvements to sociolegal status that have occurred during the beginning of the 21st century would be sufficient in contributing to better mental health among SMs. As psychological distress has increased rapidly in many societies, we may also need to incorporate our best understanding of these general changes in the prevalence of mental health problems into any future theoretical assumptions we make about how mental health among SM individuals relates to that of heterosexual individuals. We would, therefore, argue that the reasons behind, and solutions for, the remaining health disparities between heterosexual and SM individuals remain far from clear, and future research focused on both testing and expanding on existing theoretical frameworks is likely needed to improve our understanding of why SM individuals continue to experience more mental health symptoms than heterosexual individuals.

## Supporting information

Källström et al. supplementary material Källström et al. supplementary material

## Supplementary material

The supplementary material for this article can be found at http://doi.org/10.1017/S0033291725102626.
