# Supplementary material for: Mental health in sexual minorities: Change over time in a Finnish population-based sample
Source: Psychol Med. 2025 Nov 18;55:e351. doi: 10.1017/S0033291725102626 (PMC13058614; doi:10.1017/S0033291725102626)
Supplement: Källström et al. supplementary material [file S0033291725102626sup001.docx]

**Mental Health in Sexual Minorities: Change Over Time in a Finnish Population-Based Sample**

Online Supplementary Material

**Table of Contents:**

Additional Information About the Data Collection

Operationalization of Sexual Orientation

Operationalization of Gender Identity

Table S1: Patterns of Participation Across Different Time Points

Table S2: The Stability of Sexual Orientation Over Time

Table S3: Sample Sizes for the Latent Growth Curve Models

# **Additional Information About the Data Collection**

## **The T1 Data Collection**

The first wave (T1) was conducted in 2006 and targeted all Finnish-speaking twins aged 18-33, as well as their biological siblings aged 18 or older. The names and addresses of 23,577 adults fulfilling these criteria were obtained via the Central Population Registry of Finland. The potential participants were first contacted by postal mail in March 2006 with an inquiry to fill out the survey on a secure online platform or wait for a paper-based version of the survey to arrive by mail. In addition, the participants were asked about their willingness to provide a sample of saliva for DNA and hormone analyses (no further information regarding these are provided, as no results on DNA or hormone analyses are reported in this study). Participants could notify the researchers either online or via pre-paid return envelope if they did not wish to receive the questionnaire, which 958 individuals did. A reminder letter was sent at the end of July 2006 to those who had not yet responded to the questionnaire. The sample included 6,531 twins, who had a similar response rate (46%) to non-twin siblings (43%). The response rate was higher for women (57%) than for men (33%). The mean age was 24.97 years (*SD* = 4.01, range 18-33) for twins and 28.58 years (*SD* = 5.97, range 18-49) for siblings. The representativeness of the sample has been evaluated by (Johansson et al., 2013). A total of 10,524 participants (45%) responded to the survey.

## **The T2 Data Collection**

The second wave (T2) targeted all T1 participants who had stated interest in further participation. An invitation letter was sent to 2,559 men in 2012, of whom 1,173 responded to the survey on a secure online platform. In 2013, invitation letters were sent to 5,197 women, of whom 2,173 responded through the same secure online platform. The samples are described more thoroughly elsewhere (see e.g. Gunst et al., [2017] for the female sample, and Jern et al., [2013] for the male sample).

## **The T3 Data Collection**

For the third wave (T3), a total of 33,211 Finnish-speaking twins and their biological siblings aged 18 or older and residing in Finland at the time were sent invitation letters to take part in the survey online in 2018. A subset (n = 7,716) had participated at T1 and/or T2 and had indicated interest in further participation, whereas the remaining invitees were new to the study. We collected data between November 2018 and January 2019. A total of 9,564 individuals responded to the survey, resulting in a conservatively estimated response rate of 29% assuming that all invitees received the invitation letter. Of these, 9,319 individuals (97%) consented to their data being used for research purposes. One-third of the participants (30%, n = 2,755) had taken part in the T1 and/or T2 survey. For more information about the T3 data collection, please see Tybur et al. (2020).

## **The T4 Data Collection**

The fourth wave (T4) targeted Finnish twins aged 18 or older, as well as siblings (aged 18 or older) and parents of twins. Twin families and their postal addresses were obtained from the Digital and Population Data Services Agency of Finland, which maintains the National Population Registry. Addresses to 50,771 individuals were obtained from the registry in the summer of 2021. Over the period from October 2021 to February 2022, these individuals were sent an invitation letter by postal mail to participate in an anonymous online survey. Each individual was assigned a randomly generated, eight-character personal code, which they could use to log on to the survey (these randomly generated codes were subsequently used to match individuals belonging to the same family, however, so that personal information and survey responses were never combined or stored together). Individuals who did not respond in any way were sent a reminder letter 2–3 weeks after the arrival of the first invitation letter. To incentivize participation, invitees were offered the possibility to participate in a raffle for 100 gift cards worth €25 apiece to S-Ryhmä, a business conglomerate operating, for example, shops, grocery stores, hotels, restaurants and petrol stations across Finland (an individual could only win one gift card). In total, 12,269 individuals responded (24%) and of these, 854 individuals declined to give informed consent, leaving responses from 11,415 individuals. In a similar manner to the T3 data collection, a subset of participants (*n* = 3,918) had participated in one or more previous waves of the data collection. It should be noted that this response rate likely represents an underestimate. For example, according to national statistics, up to 10% of Finns move each year (https://www.stat.fi/). It is not known how many of the potential participants were not reached (e.g., due to changing addresses or death) between when the addresses were obtained from the population registry and when the invitation letters were sent out.

# **Operationalization of Gender Identity**

For descriptive statistics, we used the gender identity derived from the T4 survey. The participants received a question stating what their gender was according to the Central Population Registry (i.e., man or woman, respectively, as the Central Population Registry of Finland only recognizes two genders) and were asked to indicate whether this information was correct. Those choosing the response option corresponding to their gender in the Central Population Registry (i.e., man or woman) were classified as cisgender, whereas participants who chose another response option (i.e., “man” for registered women or “woman” for registered men, “trans man”, “trans woman”, or “other” followed by the option to specify via free-text) were classified as GM participants. Following a systematic review of the free-text answers, those who reported a cisgender identity were reclassified as cisgender (n = 4 in the T3 sample, and n = 2 in the T4 sample), and those providing unclear, inadequate, or invalid answers (e.g., “attack helicopter” or “divine being”) were excluded (n = 11 in the T3 sample, n = 6 in the T4 sample).

# **Operationalization of Sexual Orientation**

The T1 questionnaire did not ask about sexual orientation, but rather how often, on average, participants felt sexual interest towards a person of the same gender. The response options were “never”, “less than once per month”, “one to three times per month”, “once per week”, “two to three times per week”, “four to six times per week”, and “daily”. Participants who answered “never” were classified as heterosexual, whereas others were classified as SM, placing circa 75% of women and 88% of men in the heterosexual category.

At T2-T4, participants were asked to report their sexual orientation via multiple choice questions. The T2 survey provided the following sexual orientation options: “heterosexual”, “lesbian/gay”, “bisexual”, “bisexual but more attracted to women”, “bisexual but more attracted to men”, and “none of these options adequately describe my sexuality”. Participants choosing the heterosexual response option were classified as heterosexual, whereas all others were classified as belonging to a SM.

The T3 survey gave the response options of “heterosexual”, “lesbian/gay”, “bisexual”, and “other”, in which case the participants were able to specify their identity by free-text answer. Participants were classified as heterosexual if they chose the heterosexual response option, and as belonging to a SM if they chose one of the other options. Following a manual review of the free-text answers, participants reporting a heterosexual or mostly heterosexual orientation were reclassified as heterosexual (n = 24), and participants who provided unclear, inadequate, or invalid answers (e.g., “perv” or “I don’t understand the question”) were excluded (n = 5). Sexual orientation at T1, T2, and T3 was used for descriptive statistics. The T4 survey provided the response options of “heterosexual”, “lesbian/gay”, “bisexual”, “pansexual”, “asexual”, and “other”, based on which we classified those who selected heterosexual as heterosexual and all others as SM participants.

**Table S1**

*Patterns of Participation Across Different Time Points*

|  | *N of responses for each measure* | | | Time point of participation | | | |
| --- | --- | --- | --- | --- | --- | --- | --- |
| *N* | *N(BSI-18)* | *N(AUDIT-C)* | *N(SDS)* | T1 | T2 | T3 | T4 |
| 10,094 | 9,745 | 8,519 | 9,499 |  |  |  | X |
| 3,483 | 3,080 | 2,646 | 3,117 |  |  | X | X |
| 1,006 | 918 | 781 | 929 |  | X | X | X |
| 1,006 | 912 | 759 | 916 | X | X | X | X |
| 1,217 | 1,114 | 941 | 1,101 | X | X |  | X |
| 1,645 | 1,504 | 1,264 | 1,486 | X |  |  | X |
| 1,265 | 1,148 | 950 | 1,140 | X |  | X | X |
| 1,220 | 1,124 | 973 | 1,118 |  | X |  | X |

*Note.* BSI-18 = Brief Symptom Inventory 18 depression and anxiety subscales; AUDIT-C = Alcohol Use Disorders Identification Test for Consumption; SDS = Sexual Distress Scale. The numbers above reflect the sizes of the different subsamples of the present study. The first row refers to the cross-sectional subsample, where the participants had only taken part in T4. The rest of the rows display the patterns of participation among the longitudinal participants (e.g., row 2 refers to the number of participants who took part in the T3 and T4 surveys).

| **Table S2**  *The Stability of Sexual Orientation Over the Four Time Points of the Study (T1-T4)* | | | | | |
| --- | --- | --- | --- | --- | --- |
| Time points | *n^a^* | Heterosexual to Sexual Minority | | Sexual Minority to Heterosexual | |
|  |  | *n* | % | *n* | % |
| T1 🡪 T2 | 3,285 | 73 | 2 % | 527 | 16 % |
| T1 🡪 T3 | 2,751 | 29 | 1 % | 541 | 20 % |
| T1 🡪 T4 | 1,658 | 34 | 2 % | 286 | 17 % |
| T2 🡪 T3 | 1,951 | 18 | 1 % | 81 | 4 % |
| T2 🡪 T4 | 1,225 | 33 | 3 % | 43 | 4 % |
| T3 🡪 T4 | 3,524 | 173 | 5 % | 44 | 1 % |
| Total ^b^ | 14,394 | 360 | 3 % | 1,522 | 11 % |
| \| *Note.* Total *N* = 21,301. T1 = 2006. T2 = 2012-2013. T3 = 2018-2019; T4 = 2021-2022. Sexual orientation was classified via self-reported sexual interest at T1, whereas the participants of the T2, T3, and T4 data collections were asked to report their sexual orientation as an identity.  ^a^ Refers to the number of individuals who participated at both time points.  ^b^ Refers to the number of instances of participation at two time points, not unique individuals. \| \| --- \| | | | | | |

**Table S3**

*Sample Sizes for the Latent Growth Curve Models*

|  | Heterosexual | | | Sexual minority | | | |  | |
| --- | --- | --- | --- | --- | --- | --- | --- | --- | --- |
|  | *n* | *M* | *s^2^* | | *n* | *M* | *s^2^* | | Sexual minority participants |
| Anxiety |  |  |  | |  |  |  | |  |
| T1 (2006) | 1,475 | 9.43 | 14.13 | | 153 | 10.61 | 20.62 | | 9,4 % |
| T2 (2012) | 1,078 | 8.91 | 13.87 | | 120 | 9.67 | 16.87 | | 10,0 % |
| T3 (2019) | 2,740 | 10.52 | 20.81 | | 632 | 12.60 | 30.49 | | 18,7 % |
| T4 (2022) | 8,688 | 10.48 | 18.11 | | 1,057 | 13.48 | 31.42 | | 10,9 % |
| Depression |  |  |  | |  |  |  | |  |
| T1 (2006) | 1,475 | 10.66 | 18.35 | | 153 | 11.69 | 10.01 | | 9,4 % |
| T2 (2012) | 1,078 | 10.17 | 18.70 | | 120 | 10.47 | 21.57 | | 10 % |
| T3 (2019) | 2,740 | 11.87 | 25.58 | | 632 | 14.15 | 34.06 | | 18,7 % |
| T4 (2022) | 8,688 | 11.63 | 20.97 | | 1,057 | 14.56 | 32.46 | | 10,9 % |
| Alcohol use |  |  |  | |  |  |  | |  |
| T1 (2006) | 1,349 | 4.72 | 5.08 | | 139 | 4.96 | 5.14 | | 9,3 % |
| T2 (2012) | 1,007 | 4.62 | 4.85 | | 112 | 5.06 | 4.93 | | 10,0 % |
| T3 (2019) | 2,512 | 4.25 | 4.21 | | 544 | 5.47 | 4.50 | | 17,8 % |
| T4 (2022) | 7,626 | 3.94 | 4.50 | | 893 | 4.17 | 4.50 | | 10,5 % |
| Sexual Distress | | | | | | | | | |
| T1 (2006) | 1,468 | 13.74 | 27.62 | | 152 | 14.14 | 28.05 | | 9,4 % |
| T2 (2012) | 1,078 | 14.33 | 35.03 | | 120 | 14.16 | 28.45 | | 10,0 % |
| T3 (2019) | 2,821 | 14.43 | 30.62 | | 638 | 15.02 | 34.61 | | 18,4 % |
| T4 (2022) | 8,456 | 13.58 | 33.11 | | 1,043 | 14.93 | 41.78 | | 11,0 % |

*Note.* Anxiety, depression, alcohol use, and sexual distress mean levels in the samples of heterosexual and sexual minority adults used in the latent growth curve models. Anxiety and depression were measured with two dimensions from the Brief Psychiatric Scale (BSI-18), alcohol use was measured with the Alcohol Use Disorders Identification Test Consumption version (AUDIT-C) and sexual distress was measured with an abbreviated version of the Sexual Distress Scale (SDS). The data collection spanned over 16 years, including four time points (T1 = 2006, T2 = 2012, T4 = 2019, T4 = 2022).

# **References**

Gunst, A., Ventus, D., Kärnä, A., Salo, P., & Jern, P. (2017). Female sexual function varies over time and is dependent on partner-specific factors: A population-based longitudinal analysis of six sexual function domains. *Psychological Medicine*, *47*(2), 341–352. https://doi.org/10.1017/S0033291716002488

Jern, P., Piha, J., & Santtila, P. (2013). Validation of three early ejaculation diagnostic tools: A composite measure is accurate and more adequate for diagnosis by updated diagnostic criteria. *PLoS ONE*, *8*(10), e77676. https://doi.org/10.1371/journal.pone.0077676

Johansson, A., Jern, P., Santtila, P., Von Der Pahlen, B., Eriksson, E., Westberg, L., Nyman, H., Pensar, J., Corander, J., & Sandnabba, N. K. (2013). The Genetics of Sexuality and Aggression (GSA) twin samples in Finland. *Twin Research and Human Genetics*, *16*(1), 150–156. https://doi.org/10.1017/thg.2012.108

Tybur, J. M., Wesseldijk, L. W., & Jern, P. (2020). Genetic and environmental influences on disgust proneness, contamination sensitivity, and their covariance. *Clinical Psychological Science*, *8*(6), 1054–1061. https://doi.org/10.1177/2167702620951510
